# New Appliances and Things Medical

**Published:** 1897-06-19

**Authors:** 


### NEW APPLIANCES AND THINGS MEDICAL.

[We shall be glad to receive, at our Office, 28 & 29, Southampton Street, Strand, London, W.C., from the manufacturers, specimens of all new preparations and appliances which may be brought out from time to time.]

### OLD GLENLIVET WHISKEY.

(Messrs. Pattison, Leith.)

We have received a sample of Messrs. Pattison's "Special Reserve" Old Glenlivet Whiskey, and find it to be of excellent quality. Analysis shows it to be free from objectionable products, and wholesome. It is of a soft quality and fine flavour, and can be confidently recommended to whiskey consumers.

